# Anemia as a risk factor for childhood asthma

**DOI:** 10.4103/0970-2113.63605

**Published:** 2010

**Authors:** K. Ramakrishnan, Ashwin Borade

**Affiliations:** *Department of Pediatrics, Amrita School of Medicine, Kochi, India*

**Keywords:** Anemia, childhood asthma, lower respiratory tract infections

## Abstract

**Objective:**

This prospective-(cohort) study was conducted to evaluate whether anemia is a risk factor for childhood asthma.

**Materials and Methods:**

Two hundred children in the age group of 2-18 years who attended the Outpatient Department with upper respiratory / lower respiratory tract infections were included in this study. One hundred children with anemia were taken as the study group and another 100, age - and sex-matched children without anemia were taken as the control.They were subjected to complete blood count (CBC) C-reactive protein (CRP) estimation, Mantoux test and chest X-ray. Pulmonary function tests (PFTs) were performed on those above six years showing evidence of asthma. Peripheral smear, serum ferritin and serum iron-binding capacity were estimated for all anemic children.

**Results:**

Asthma was present in 74 (74%) children in the study group and in 33 (33%) children in the control group. Iron-deficiency anemia was present in 85 (85%) anemia of chronic infection in 20 (20%) and the other five (5%) had hemolytic anemia. Anemia was found to be a risk factor for childhood asthma.

**Conclusion:**

Anemic children were 5.75 times more susceptible to asthmatic attacks when compared with nonanemic children.

## INTRODUCTION

Asthma is a common medical problem encountered by clinicians dealing with children. Its incidence has substantially increased worldwide.[1] It is a major cause of morbidity and mortality among the pediatric age group.[2] It is a chronic inflammatory condition of the lung airways resulting in episodic airflow obstruction.[3]

Anemia associated with acute infections occurs more commonly in children than in adults. Iron deficiency exerts adverse effects on immune response and alters the metabolism and growth of pathogens. It has already been reported that low hemoglobin impaires tissue oxygenation and acts as an independent risk factor for developing lower respiratory tract infections (LRTI) in children.[4] There are very few reports available in medical literature regarding the association of anemia and childhood asthma. Since anemia has been shown to be a risk factor for LRTI,[4], this study was taken up to see whether any relationship exists between anemia and childhood asthma.

## MATERIALS AND METHODS

This prospective (cohort) study was conducted in 200 children in the age group of 2-18 years, who attended the out-patient unit of the Department (OPD) of Pediatrics, Amrita School of Medicine, Kochi, Kerala, India, from April 2003 to March 2005. Hundred children with anemia having upper respiratory tract (URI) or lower respiratory tract (LRTI) infections and another age- and sex- matched control group of 100 non-anemic children, who came to the OPD for URI/LRTI were taken as control. Those children were followed up for a period of three years at monthly intervals. Any incidence of wheeze during the period was noted.

Inclusion criteria: Upper respiratory tract consists of the nasal cavity, pharynx, and ends at the level of vocal cord. Hence rhinitis, sinusitis, pharyngitis, pharyngotonsillitis, epiglottitis and laryngitis were included.LRTI: The lower respiratory tract consists of the trachea, bronchi, bronchioles, and alveoli. Acute bronchitis, bronchopneumonia and lobar pneumonia were considered as LRTI.

Children with the following criteria were exempted from this study:(i) Congenital malformations of the chest wall (ii) severe systemic illness, and (iii) Protein Energy Malnutrition (PEM) > Grade III as per Indian Academy of Pediatrics (IAP).[5]

A detailed history was taken and a thorough clinical examination was conducted each and every patient. They were subjected to a battery of investigations, which included complete blood count (CBC), C - reactive protein estimation (CRP), Mantoux test, and chest X-ray. X-ray chest was taken by conventional methods and interpreted as described.[6] Weight was recorded for all children to assess the nutritional status. Peripheral smear, serum ferritin and serum iron binding capacity were done in all anemic children. All children above six years, having clinical evidence of asthma were subjected to pulmonary function tests (PFTs).

A child was considered anemic if the hemoglobin (Hb) level was below 11 g/dL.[7]

Asthma was diagnosed by clinical examination and by applying the following criteria (i) episodic symptoms of airflow obstruction, (ii) more than three episodes were present, (iii) airway obstruction was at least partially reversible and (iv) alternative diagnosis were excluded.[8] The results were statistically analyzed.

### Statistical analysis

Numerical variables were reported in terms of mean and standard deviations. Categorical variables were reported in terms of numbers and percentages.

The association of each of the categorical variables with the response variable was assessed by The Chi-square test and the strength of their association were computed by an unadjusted Odds ratio. Variables showing a statistically significant association in a univariate analysis with the outcome variable up to *P* = 0.25 were considered as risk factors. Only those variables were explored to find the risk factor for asthma. In a multivariate analysis variables showing *P* value less than 0.05 were considered to be statistically significant.

## RESULTS

Two hundred children in the age group of 2-18 years were studied. CRP was more than 6 mg/dL in 44 children (44%) in the study group and 13 (13%) in the control group. Mantoux test was positive in four (4%) in the study group and three (3%) in the control group. Hyperinflated lung fields were seen in 28 children (28%) in the study group at the time of initial examination and 10 in the control group. In both groups, age was not found as a significant factor affecting the result. *P* = 0.38 and 0.47 respectively for the study group and control group. Seventy four children (74%) in the study group and 33 (33%) in the control group had asthmatic attacks during the follow -up period. Among the anemic group 85 (85%) had iron- deficiency anemia (IDA) 10 had anemia of chronic infection and the other five (5%) had hemolytic anemia (HA). Descriptive data regarding age, sex, mean, and standard deviation of the number of asthmatic children are shown in Tables 1‐3.

**Table 1:** Bivariate relationship between patient characteristics and asthma

Variables	Study cases (n=100)	Controls (n=100)	Unadjusted odds ratio	95% CI	*P* value
Age (years)					
<6	65	64
6-14	32	34
>14	3	2
Sex
M	63	58	1.00
F	37	42	1.23	0.67-2.26	0.47
Asthma
Yes	74	33	5.78	0.000
No	26	67	1.00	3.01-11.19	(Sig)

CI: Confidence Interval, Sig: Statistically significant, NS: Not significant

**Table 2:** Multivariate logistic regression analysis showing the risk factor of asthma

Risk Factor	Adjusted OR	95% Lower	CI Upper	*P* value
Anemia	5.750	2.989	11.060	0.000

CI: Confidence Interval, OR: Odds ratio

**Table 3:** Shows average number of wheezing episodes/year and family history of atopy

Study group (n = 100) Average no. of wheezing episodes/ year		Family h/o atopy	Control group (n = 100) Average no. of wheezing episodes/ year	Family h/o atopy
Age				
<6	16	10(15)	4	11(17)
6-14	10	6(18)	3	7(21)
>14	6	1(33)	2	Nil (0)
Total	17	17	18	(18)

h/o: History of, no: Number, Figures in parenthesis are in percentage

## DISCUSSION

Asthma has gained much interest in critical care illness in recent years following many studies, because of increased case prevalence, morbidity and for a better understanding of the pathogenesis.

In our study the incidence of asthmatic attacks were more in the study group compared to the controls. The lowest Hb was 5.3 gm/dL with a mean of 9.9 gm/dL. Among the anemic group 50 (50%) had moderate asthma 10 (10%) had mild persistent asthma and the remaining 14 (14%) had mild intermittent asthma. In the control group 33 (33 %) had asthmatic attacks.

We did not find any predominance for age or sex with regard to the incidence. Male predominance had been reported in a study.[9]

There are few reports available in literature regarding the relationship of anemia with childhood asthma. Some are of the opinion that iron supplements significantly reduces the morbidity of upper respiratory tract infection (URTI) in children.[10] An increased incidence of anemia has been reported in chronic obstructive pulmonary disease (COPD).[11]

The increased incidence of asthmatic attacks in anemic children may be due to the following facts: Hemoglobin facilitates oxygen (O_2_) and carbon dioxide transport. It carries and inactivates nitric oxide (NO) and also plays the role of a buffer.[12] Hemoglobin in the blood is mainly responsible for stabilizing the oxygen pressure in the tissues.[13] Qualitative and/or quantitative reduction in Hb may adversely affect the normal functions.

Despite the limitations, our study highlights the role of anemia in triggering an asthmatic attack.

### Limitations of study

This study certainly has some limitations. Being a tertiary care center, many of the cases were referred ones. Therefore the patient profile probably does not truly reflect the prevalence of a particular disease in the representative population. Only 200 children were studied. Hence a definite conclusion cannot be made based on this study alone. A large number of patients has to be studied to confirm our findings

## CONCLUSION

It is a significant finding that anemic children are 5.75 times more susceptible to develop child hood asthma compared to the non-anemic group.
